# Variants of Interleukin-22 Gene Confer Predisposition to Autoimmune Thyroid Disease

**DOI:** 10.1155/2017/3428236

**Published:** 2017-08-03

**Authors:** Rong-hua Song, Qian Li, Wen Wang, Qiu-ming Yao, Xiao-qing Shao, Jin-an Zhang

**Affiliations:** Department of Endocrinology, Jinshan Hospital of Fudan University, No. 1508 Longhang Road, Jinshan District, Shanghai 201508, China

## Abstract

As there are no previous studies on the interleukin-22 (IL-22) variants in autoimmune thyroid disease (AITD), the present study aimed to explore the association between polymorphisms of IL-22 and the predisposition to AITD. The study had 975 AITD patients, including 639 Graves' disease (GD) and 336 Hashimoto's thyroiditis (HT) individuals and 851 healthy cohorts. Ligase detection reaction (LDR) and direct sequencing method were used for genotyping the IL-22 gene polymorphisms at rs2046068, rs2227478, rs2227485, rs11611206, and rs1179251. In comparison to female controls, genotype CC of rs1179251 was increased in the female AITD patients. Alleles C at rs2046068, C at rs2227478, and C at rs1179251 linked to the susceptibility of HT males. Genotype CC in rs1179251 was higher in male HT. Variants at rs2046068, rs2227478, and rs1179251 were associated with the AITD teenagers. Besides, genotype GG in rs11611206 was correlated with thyroid-associated ophthalmopathy (TAO). Moreover, allele G at rs11611206 was associated with decreased risk for TAO by 28.9%. Similarly, genotype CC of rs1179251 and genotype GG of rs11611206 were associated with Graves' ophthalmopathy (GO). Allele G in rs11611206 increased people with HT towards the predisposition of hypothyroidism. In conclusion, genetic variants of IL-22 are associated with the occurrence of AITD.

## 1. Introduction

Autoimmune thyroid disease (AITD), known to be the most common endocrine autoimmune disease, is an organic disease caused by immune irregulation related to the thyroid gland. AITD mainly includes two subtypes, Graves' disease (GD) and Hashimoto's thyroiditis (HT). The prevalence of AITD in the whole population has reached 2%–5% [1]. The presence of autoimmune antibodies, including antibody against TSH receptor (TRAb), anti-TPO antibody (TPOAb), and thyroglobulin antibody (TGAb), is the key immunological feature of AITD. AITD is also an immune polygenic disorder with a strong genetic component [2]. Recent advances in understanding the genetic basis for AITD documented the potential involvement of several genes in its pathophysiology. Polymorphisms in the genes, like TNFAIP3 [2, 3], IL-37 [4], and IRAK1/MECP2 [5], have been implicated in the occurrence and development of AITD.

Interleukin-22 (IL-22), belonging to the IL-10 cytokine family, is mainly expressed and secreted by Th22 (CD4 + IL22+) cells [6]. IL-22 gene polymorphisms have been found to be a risk factor for various autoimmune diseases, like psoriasis vulgaris [7] and celiac disease [8]. Recently, a study of ours revealed the protein level of interleukin-22 (IL-22) in serum, and the expression of IL-22 mRNA in peripheral blood mononuclear cells (PBMCs) was elevated in GD patients compared to healthy volunteers, implying IL-22 as an inflammatory cytokine participating in GD [9]. However, the relationship between IL-22 polymorphisms and AITD as yet remains unknown.

The present study was focused on investigating the allele frequencies, genotype distributions of the IL-22 variants, and their correlation with the development of AITD and its clinical phenotypes.

## 2. Material and Methods

### 2.1. Ethics Statement

This study was approved by the institutional review board of Jinshan Hospital of Fudan University. The importance of the research was explained to all participants and written informed consent was obtained from each participant prior to the research.

### 2.2. Design and Setting

A case-control pilot was performed to define the allelic and genotypic profiles of polymorphisms (rs2046068, rs2227478, rs2227485, rs1179251, and rs11611206) within the IL-22 region in autoimmune thyroid disease in a cohort of Chinese origin and subphenotype groups.

### 2.3. Study Participants

In total, all the subjects (including established AITD patients and controls) were local ethnic Han Chinese. The study group enrolled 975 patients first diagnosed with AITD, including 639 Graves' disease and 336 Hashimoto's thyroiditis ones, and 851 healthy volunteers. The inclusion criterion to the AITD was consistent with that as detailed in our previous papers [5, 10]. GD diagnosis was based on clinical manifestations, laboratory assessments (hyperthyroidism and positive TRAb, with or without positive TPOAb or TgAb), and diffuse goiter of the thyroid; HT was diagnosed based on the positive of either TPOAb or TgAb, with or without clinical and biochemical hypothyroidism and the presence of an enlarged thyroid. The suspected HT patients were further confirmed by the more accurate fine needle aspiration biopsies. The criterion for diagnosis of euthyroid in HT patients is normal TSH, FT3, and FT4. All AITD patients were collected from the Out-patient Clinic of Endocrinology of Jinshan Hospital of Fudan University. The sex- and age-matched controls were from the Healthy Check-up Center of the same hospital, and they had no sibship with individuals in the patient group. The exclusion criteria for control subjects were positive thyroid antibodies against TPO tested by the immunochemiluminescence method (Roche Company, Shanghai, China) with high specificity and sensitivity. All the clinical and laboratory parameters were collected from patients' files and then analyzed.

### 2.4. Genotyping

Genomic DNA from AITD patients and healthy volunteers was extracted from 2 ml of peripheral whole blood using commercial RelaxGene Blood DNA System provided by Tiangen Biotech Company (Beijing, China), in accordance with the manufacturer's protocol. All DNA samples measured the concentration and A260/A280 ratio by NanoDrop 2000 Spectrophotometer provided by Thermo Scientific Company (USA). Only the DNA samples with great purity and concentration were prepared for the next polymorphism genotyping.

In the present study, marker-tagging single-nucleotide polymorphisms (SNPs) which mapped the IL-22 gene were chosen from the previous studies [7, 8, 11]. At the same time, we chose marker-tagging SNPs from the Hapmap CHB data using the software named HaploView to meet the following criteria: minor allele frequency (MAF) >0.05, Hardy-Weinberg equilibrium (HWE) with *p* > 0.01, and logarithm of odds (LOD) >3.0. For the IL-22 gene, we finally determined five loci covering the whole region of the gene to capture all the most common variants. In final, five tagSNPs, rs2046068, rs2227478, rs2227485, rs1179251, and rs11611206 of the IL-22 region, were selected. Genotyping was conducted using the ligase detection reaction (LDR) platform according to the manufacturer's instruction. Positive controls (duplication of the same DNA samples) and blank controls (blank samples without DNA) were both used in SNP genotyping to further ensure the reliable detection quality. All genotype results were recorded manually by different persons and inconsistent results were regenotyped by direct sequencing. Only SNPs that passed the 98% quality control threshold were subjected to the next statistical analyses. The SNPs with allele frequencies meeting Hardy-Weinberg equilibrium were included for next analysis. Specific primers of the five loci in the IL-22 gene are displayed as follows:

- rs2046068: forward primer-AAAAGGCAACTCAGGTTGCG,

reverse primer-CTCCCCTCAACAACTTAGAC;

(ii) rs2227478: forward primer-TGAGATGGCACAGACCTAAG,

reverse primer-TCTGGCCACCTTCACAAATG;

(iii) rs2227485: forward primer-GTTTTGTCTTAGTAGAGTTC;

reverse primer- TGAGTCCGTGACCAAAATGC;

(iv) rs11611206: forward primer-CACCTGTGAATCTCAGTTCC,

reverse primer-TCATGCTAGAGACCTGACAC;

(v) rs1179251: forward primer-GGTTGGGATCTTAGCTTGTC;

reverse primer-ACCTGCATTCTAGCCCTATC.

### 2.5. Clinical Subphenotype Analysis

Clinical subphenotype characteristic analyses were conducted by the case-only approach, in which basic allelic and genotypic examinations were performed by comparing minor allele and genotype frequency of cases with a specific subphenotype to the whole case group. The clinical subphenotypes include (1) onset age of the disease (≤18 years old versus ≥19 years old); (2) with or without thyroid eye disease, patients with mild or more serious than mild (moderate to severe) orbitopathy were diagnosed as ophthalmopathy according to EUGOGO criteria [12]; (3) with or without hypothyroidism in the HT group.

### 2.6. Statistical Analysis

All of the data were mainly tested with the SPSS Statistics 20.0 software (IBM, Armonk, NY). Linkage disequilibrium (LD) test and haplotype frequency calculation were carried on using the software of HaploView 4.2. Evaluation of Hardy-Weinberg equilibrium (HWE) of the five loci using the chi-square (*χ*^2^) test was done prior to performing an association statistics. And exploration of the association was performed by comparing allele and genotype between AITD subjects and healthy ones using the *χ*^2^ test or Fisher's exact analysis. Linkage disequilibrium (LD) analysis was performed for the five loci using the HaploView program. Two-tailed *P* values <0.05 were considered of statistical significance in all assessments. Odds ratio (OR) was to assess the correlation between each locus and AITD.

## 3. Results

### 3.1. Characteristics of Chinese AITD and Control Subjects

Table 1 showed all the phenotype information about our cohort. We studied 975 patients with AITD (639 GD cases and 336 HT cases) and 851 healthy volunteers of Chinese origin. In the AITD group, there were 742 female and 233 male patients, and the average age of the patients was 36.210 ± 14.345 years old. The median age at diagnosis was 33.340 ± 14.160 years old (range 1–77 years). There were 202 subjects with family history in the AITD group with a percentage of 20.72%. Additionally, there were 105 teenagers in this case group with a percentage of 10.77%. The percentages of family history were 30.48% in those with onset age ≤18 years old and 12.41% in those subjects with onset age ≥19 years old. Besides, we unraveled that there were 118 AITD patients with ophthalmopathy (with a percentage of 12.10%), including 112 GD patients. TRAb were positive in all GD patients. In addition, in the HT subgroup, 171 subjects had hypothyroidism (at a rate of 50.89%), 79.53% cases were with positive TPOAb (267 ones), and 52.30% cases were with positive TGAb (176 ones). In the control group, there were 567 female and 284 male patients and the average age was 38.700 ± 8.945 years old. No statistical significance was found in comparing the age and gender in the AITD, GD, and HT groups to the control group (*P* > 0.05, data not shown).

### 3.2. Hardy-Weinberg Equilibrium Analysis

IL-22 SNP genotyping identified the genotypes A/A, A/C, and C/C at rs2046068; T/T, T/C, and C/C at rs2227478; C/C, C/T, and T/T at rs2227485; C/C, C/G, and G/G at rs1179251; and G/G, G/A, and A/A at rs11611206. According to the genotyping results, the distributions of all genotypes at rs2046068, rs2227478, rs2227485, rs1179251, and rs11611206 in the IL-22 gene in the control group met the expectations of Hardy-Weinberg equilibrium (*P* > 0.01). Besides, we detected linkage disequilibrium (LD) between any two loci in the five ones of IL-22. The *D* values between any two loci among the four SNPs (rs2046068, rs2227478, rs2227485, and rs1179251) were obtained larger than 0.9, which shows that the four loci were in strong linkage disequilibrium.

### 3.3. Allelic and Genotypic Analysis

There was no significant association between the IL-22 gene polymorphisms (rs2046068, rs2227478, rs2227485, rs1179251, and rs11611206) and AITD, GD, and HT (*P* values all larger than 0.05), which were shown in Table 2. But the frequency of allele A of rs2046068 (77.83%) in HT patients was lower than that in controls (81.02%), although this association failed to reach significant probabilities (*P* = 0.079). Moreover, as seen in Table 3, locus analysis of the alleles and genotype distribution in female AITD patients and female controls indicated that genotype CC of rs1179251 linked to the susceptibility of AITD (*P* = 0.048). But we found no evidence for an association between the allele and genotype distribution and the risk of AITD in male patients (*P* > 0.05, shown in Table 3).

Similarly, there were no differences in the allele and genotype distributions in male GD and female GD versus those in corresponding controls (*P* values all larger than 0.05, data not shown). Nevertheless, as displayed in Table 4, compared to that in male controls, allele C at rs2046068, allele C at 2227478, and allele C at 1179251 can increase the risk towards the predisposition of HT in males (*P* = 0.0.27, *P* = 0.031, and *P* = 0.039, resp.). The difference in allele frequency observed for the polymorphism in rs1179251 was further verified by genotype probabilities, and genotype CC of rs1179251 was also associated with HT in male subjects (*P* = 0.007).

### 3.4. Genotyping-Phenotype Correlations

When next comparing the allele and genotype distributions of IL-22 polymorphisms the onset age between ≤18 years old and ≥19 years old, we found that genotype A/C at rs2046068, T/C at rs2227478, and C/G at rs1179251 were associated with the onset age in teenagers. Among them, A/C at rs2046068 and T/C at rs2227478 linked to the AITD adolescents (*P* = 0.034 and *P* = 0.028, resp.). Besides, the frequency of allele C at rs1179251 in AITD youngsters was more than that in AITD adults (*P* = 0.039, OR = 1.397). We further analyzed the allele and genotype frequencies of IL-22 SNPs with ophthalmopathy in AITD patients, and this study demonstrated that the SNP rs11611206 within the IL-22 gene was associated with thyroid-associated ophthalmopathy (TAO) (*P* = 0.023). Furthermore, the allele G in rs11611206 can decrease the risk of TAO by 28.9% (*P* = 0.097). The above data were all shown in Table 5.

As shown in Table 6, the similar stratification analysis method was conducted in the subgroup in GD and HT. Similarly, the genotypes CC in rs1179251 and GG in rs11611206 were correlated with Graves' ophthalmopathy (GO) (*P* = 0.036 and *P* = 0.045, resp.). In hypothyroidism patients with HT versus euthyroidism subjects with HT, the results showed the correlation with G allele in rs11611206 (*P* = 0.024, OR = 1.828).

### 3.5. Haplotype Analysis

**Figure 1:**
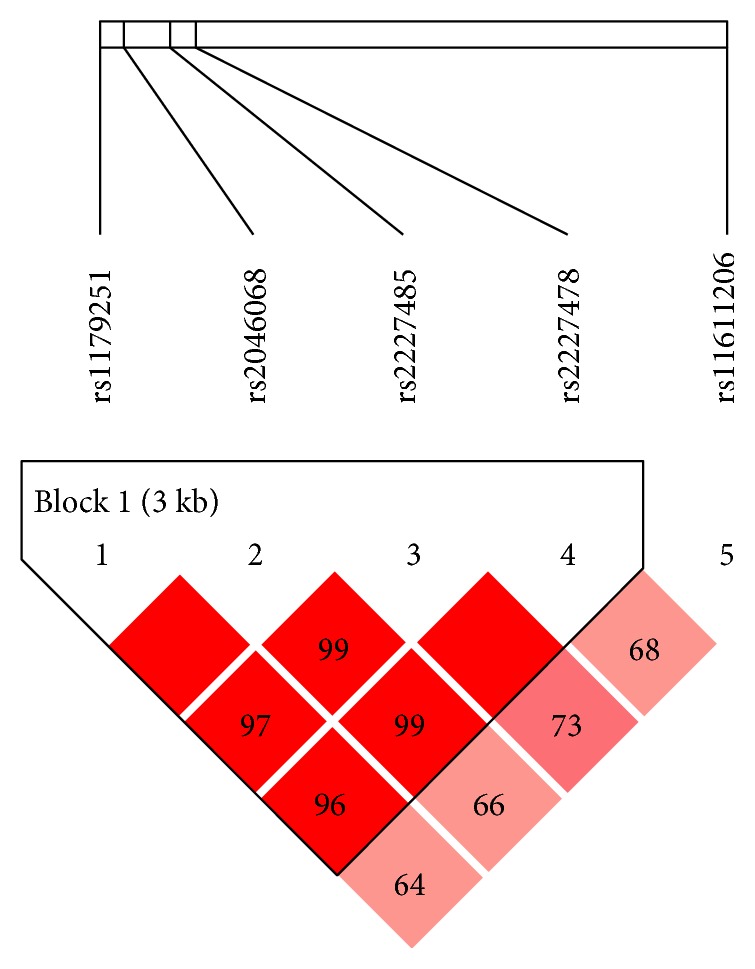
Haplotype by HaploView software analyses.

Figure 1 showed that haplotype analysis performed for the 5 SNPs revealed that only one block exists including rs2046068, rs2227478, rs2227485, and rs1179251.  Table 7 displayed that there were three main haplotypes, namely, CATT, GACT, and CCCC, of these four loci. Through HaploView software examination, there was no association between those three haplotypes and AITD, GD, and HT (*P* > 0.05).

## 4. Discussion

Recently, it is clear that AITD has become one of the most important autoimmune healthcare in the population. However, the pathogenesis of AITD remains elusive. As we all know, the occurrence of AITD is with obvious sex tendency, and this disease is more popular in women with the ratio of female to male being 5 : 1 to 10 : 1 [13]. The disease also displays a family aggregation phenomenon, as there are 40%–50% of patients with their families suffering from thyroid disease [14]. Increasing data have demonstrated that environment factors, immune elements, and genetic susceptibility are all involved in the etiology of this disease [15, 16].

IL-22, as a member of IL-10 cytokine superfamily, is mainly secreted by Th22 cells and also by their dominant functional cytokine [17]. Our precious publication about a functional study to explore the possible biological mechanisms underlying the association between IL-22 and AITD has indicated that serum IL-22, the mRNA expression of IL-22, and Th22 cells are all increased in GD patients, implying that IL-22 as a proinflammatory factor participates in onset and development of AITD [9]. Recent studies have demonstrated associations between several variants of IL-22 and multiple immune-related diseases [7, 8, 11, 18]. In the present study, we attempted to find out the potential link of polymorphisms of IL-22 with the risk of AITD.

There were no significant differences among the five IL-22 gene polymorphism distribution in the whole AITD, GD, and HT subjects and controls, except that there was a weak association between allele A of rs2046068 and HT patients (although with no statistical significance). Because of the gender trend in AITD [13], we subsequently conducted the sex-stratified analysis and indeed found some interesting and significant results through gender-stratified comparison. The results of our case-control study discovered that several loci of IL-22 are associated with the susceptibility of AITD in the Han Chinese population. For instance, genotype GG in rs1179251 linked to the female AITD and allele A in rs2046068 and allele T in rs2227478 decreased the risk to HT by 44.3% and 43.6%, respectively, and allele C in rs1179251 increased the predisposition for HT by 73.9% in male patients. The current study and our precious report [9] both imply that IL-22 participates in the pathogenesis of AITD, through polymorphisms and aberrant expression. There is no doubt that IL-22 as a vital cytokine takes part in the immune dysfunction of AITD, and as a key gene member, it will be added to the list of genes which influences the predisposition for AITD.

Subsequent phenotype analyses further confirmed the association between IL-22 variants and AITD subphenotypes. In teenagers, the onset of AITD is often with much obvious family history [14], which was also identified in our study that showed that the percentage of family history in teenager patients (30.48%) was higher than that in adult patients (12.41%). Our current research showed that genotype AA in rs2046068 and genotype TT in rs2227478 were correlated with the risk towards teenagers to develop AITD. Besides, it showed that allele C of rs1179251 increases the teenagers' risk for AITD by 39.7%. Similarly, a pervious paper of ours also reported that there is an association between TNFSF4 gene variations and AITD adolescents [19]. So these results strongly suggest that the occurrence of thyroid dysfunction in teenagers can be caused by family history, which means the genetic background. The eye disorder associated with AITD, called thyroid-associated ophthalmopathy, drastically reduces the quality of life in affected patients and even contributes to the disability of patients [20]. Enormous amounts of documents have shown that TAO is a complex disease with multifaceted mechanism including smoking history, thyroid dysfunction, and positive TRAb [21, 22]. We showed for the first time that genotype GG in rs11611206 within IL-22 gene is associated with TAO. When it comes to TAO, most people think first of Graves' orbitopathy (GO), as more than 80% TAO patients also present with Graves' disease [23]. In our present study, as for GO, the SNPs in IL-22 at both rs1179251 (genotype CC) and rs11611206 (genotype GG) linked to GO. Autoimmune hypothyroidism is largely caused by Hashimoto's thyroiditis, in which irregulatory immune responses against to the thyroid tissue happens [24]; therefore, evaluation of thyroid function in HT is of particular importance. Our results suggested that rs11611206 polymorphism of IL-22, including both allele G and genotype GG, may be a genetic risk factor for autoimmune hypothyroidism in HT. Thus polymorphisms in the IL-22 gene confer predisposition to some subclasses of AITD.

This is the first report about the IL-22 gene polymorphisms in AITD. Also, this is the first study on the link between the loci in the IL-22 region and AITD susceptibility in Chinese population. Further case-control researches with larger populations and more races are needed to identify these observations.

## 5. Conclusion

Current findings indicated that polymorphisms in IL-22 may explain part of the AITD genetic tendency. To be sure, IL-22 is a risk factor for the development of AITD.

## Figures and Tables

**Table 1:** Clinical characteristics of AITD patients and controls.

Clinical phenotype	AITD (%)	GD (%)	HT (%)	Control (%)
Number	975	639	336	851
Gender				
Male	233 (23.90)	190 (29.73)	43 (12.80)	284 (33.37)
Female	742 (76.10)	449 (70.27)	293 (87.20)	567 (66.63)
Average age	36.210 ± 14.345	—	—	38.700 ± 8.945
≤18 years	105 (10.77)	—	—	—
With family history	32 (30.48)	—	—	—
≥19 years	870 (89.23)	—	—	—
With family history	108 (12.41)	—	—	—
Age at diagnosis	33.340 ± 14.160	—	—	—
Ophthalmopathy (+)	118 (12.10)	112 (17.53)	6 (1.79)	—
Family history (+)	202 (20.72)	—	—	—
Smoking history (+)	291 (29.85)	—	—	—
TRAb (+)	—	639 (100.00)	—	—
TPOAb (+)	—	—	267 (79.53)	—
TGAb (+)	—	—	176 (52.30)	—
Hypothyroidism	—	—	171 (50.89)	—

AITD: autoimmune thyroid disease; GD: Graves' disease; HT: Hashimoto's thyroiditis.

**Table 2:** Comparison of allele and genotype frequencies of IL-22 SNPs in AITD, GD, and HT patients and controls.

SNP ID	Groups				*P* value (OR/95% CI)		
	NC (%)	AITD (%)	GD (%)	HT (%)	AITD versus NC	GD versus NC	HT versus NC
rs2046068							
AA	559 (65.69)	622 (63.79)	419 (65.57)	203 (60.42)	0.495	0.67	0.211
CC	31 (3.64)	45 (4.62)	29 (4.54)	16 (4.76)			
AC	261 (30.67)	308 (31.59)	191 (29.89)	117 (34.82)			
A	1379 (81.02)	1552 (79.59)	1029 (80.52)	523 (77.83)	0.278 (0.913/0.775–1.076)	0.729 (0.968/0.805–1.163)	0.079 (0.822/0.661–1.023)
C	323 (18.98)	398 (20.41)	249 (19.48)	149 (22.17)			
rs2227478							
TT	558 (65.57)	622 (63.80)	418 (65.42)	204 (60.72)	0.609	0.828	0.271
CC	32 (3.76)	44 (4.51)	28 (4.38)	16 (4.76)			
TC	261 (30.67)	309 (31.69)	193 (30.20)	116 (34.52)			
T	1377 (80.90)	1553 (79.64)	1029 (80.52)	524 (77.98)	0.339 (0.923/0.784–1.087)	0.790 (0.975/0.812–1.172)	0.108 (0.836/0.671–1.040)
C	325 (19.10)	397 (20.36)	249 (19.48)	148 (22.02)			
rs2227485							
CC	246 (28.91)	315 (32.31)	200 (31.30)	115 (34.23)	0.219	0.508	0.162
TT	196 (23.03)	227 (23.28)	150 (23.47)	77 (22.92)			
CT	409 (48.06)	433 (44.41)	289 (45.23)	144 (42.85)			
C	901 (52.94)	1063 (54.51)	689 (53.91)	374 (55.65)	0.341 (1.065/0.935–1.214)	0.598 (1.040/0.899–1.203)	0.232 (1.116/0.932–1.335)
T	801 (47.06)	887 (45.49)	589 (46.09)	298 (44.35)			
rs1179251							
CC	354 (41.60)	427 (43.79)	278 (43.51)	149 (44.35)	0.179	0.226	0.432
GG	85 (9.99)	115 (11.80)	77 (12.05)	38 (11.31)			
CG	412 (48.41)	433 (44.41)	284 (44.44)	149 (44.34)			
C	1120 (65.80)	1287 (66.00)	840 (65.73)	447 (66.52)	0.901 (1.009/0.879–1.157)	0.965 (0.997/0.855–1.161)	0.741 (1.032/0.855–1.247)
G	582 (34.20)	663 (34.00)	438 (34.27)	225 (33.48)			
rs11611206							
GG	686 (80.61)	782 (80.21)	506 (79.19)	276 (82.14)	0.298	0.342	0.273
AA	4 (0.47)	11 (1.12)	7 (1.09)	4 (1.19)			
GA	161 (18.92)	182 (18.67)	126 (19.72)	56 (16.67)			
G	1533 (90.07)	1746 (89.54)	1138 (89.05)	608 (90.48)	0.596 (0.944/0.761–1.170)	0.364 (0.896/0.707–1.135)	0.765 (1.047/0.774–1.417)
A	169 (9.93)	204 (10.46)	140 (10.95)	64 (9.52)			

SNP: single-nucleotide polymorphism; AITD: autoimmune thyroid disease; GD: Graves' disease; HT: Hashimoto's thyroiditis; NC: normal controls; OR: odds ratio; CI: confidence interval.

**Table 3:** Allele and genotype distribution of IL-22 SNPs in different sex groups of AITD patients.

Loci	Male				Female			
	NC (%)	AITD (%)	*P* value	OR/95% CI	NC (%)	AITD (%)	*P* value	OR/95% CI
rs2046068								
AA	195 (68.66)	147 (63.09)	0.230		364 (64.20)	475 (64.02)	0.955	
CC	7 (2.47)	11 (4.72)			24 (4.23)	34 (4.58)		
AC	82 (28.87)	75 (32.19)			179 (31.57)	233 (31.40)		
A	472 (83.10)	369 (79.18)	0.108	0.774/0.566–1.058	907 (79.98)	1183 (79.72)	0.867	0.984/0.811–1.193
C	96 (16.90)	97 (20.82)			227 (20.02)	301 (20.28)		
rs2227478								
TT	195 (68.66)	148 (63.52)	0.329		363 (64.02)	474 (63.88)	0.982	
CC	8 (2.82)	11 (4.72)			24 (4.23)	33 (4.45)		
TC	81 (28.52)	74 (31.76)			180 (31.75)	235 (31.67)		
T	471 (82.92)	370 (79.40)	0.148	0.794/0.580–1.086	906 (79.89)	1183 (79.88)	0.901	0.989/0.816–1.199
C	97 (17.08)	96 (20.60)			228 (20.11)	301 (20.28)		
rs2227485								
CC	76 (26.76)	64 (27.47)	0.880		170 (29.98)	251 (33.83)	0.171	
TT	70 (24.65)	53 (22.74)			126 (22.22)	174 (23.45)		
CT	138 (48.59)	116 (49.79)			271 (47.80)	317 (42.72)		
C	290 (51.06)	244 (52.36)	0.676	1.054/0.825–1.346	611 (53.88)	819 (55.19)	0.505	1.054/0.903–1.231
T	278 (48.94)	222 (47.64)			523 (46.12)	665 (44.81)		
rs1179251								
CC	119 (41.90)	104 (44.63)	0.449		235 (41.45)	323 (43.53)	0.048^∗^	
GG	31 (10.92)	18 (7.73)			54 (9.52)	97 (13.07)		
CG	134 (47.18)	111 (47.64)			278 (49.03)	322 (43.40)		
C	372 (65.49)	319 (68.45)	0.314	1.143/0.881–1.484	748 (65.96)	968 (65.23)	0.696	0.968/0.823–1.139
G	196 (34.51)	147 (31.55)			386 (34.04)	516 (34.77)		
rs11611206								
GG	232 (81.69)	184 (78.97)	0.602		454 (80.07)	598 (80.59)	0.387	
AA	1 (0.35)	2 (0.86)			3 (0.53)	9 (1.21)		
GA	51 (17.96)	47 (20.17)			110 (19.40)	135 (18.20)		
G	515 (90.67)	415 (89.06)	0.391	0.837/0.558–1.256	1018 (89.77)	1331 (89.69)	0.946	0.991/0.768–1.279
A	53 (9.33)	51 (10.94)			116 (10.23)	153 (10.31)		

^∗^ *P* < 0.05. SNP: single-nucleotide polymorphism; AITD: autoimmune thyroid disease; NC: normal controls; OR: odds ratio; CI: confidence interval.

**Table 4:** Allele and genotype analysis of IL-22 SNPs in male patients with HT.

Loci	Male			
	NC (%)	HT (%)	*P* value	OR/95% CI
rs2046068				
AA	195 (68.66)	22 (51.16)	0.084	
CC	7 (2.47)	2 (4.65)		
AC	82 (28.87)	19 (44.19)		
A	472 (83.10)	63 (73.26)	0.027^∗^	0.557 (0.329–0.942)
C	96 (16.90)	23 (26.74)		
rs2227478				
TT	195 (68.66)	22 (51.16)	0.086	
CC	8 (2.82)	2 (4.65)		
TC	81 (28.52)	19 (44.19)		
T	471 (82.92)	63 (73.26)	0.031^∗^	0.564 (0.334–0.954)
C	97 (17.08)	23 (26.74)		
rs2227485				
CC	76 (26.76)	11 (25.58)	0.984	
TT	70 (24.65)	11 (25.58)		
CT	138 (48.59)	21 (48.84)		
C	290 (51.06)	43 (50.00)	0.855	0.959 (0.609–0.509)
T	278 (48.94)	43 (50.00)		
rs1179251				
CC	119 (41.90)	23 (53.49)	0.007^∗^	
GG	31 (10.92)	0		
CG	134 (47.18)	20 (46.51)		
C	372 (65.49)	66 (76.74)	0.039^∗^	1.739 (1.024–2.952)
G	196 (34.51)	20 (23.26)		
rs11611206				
GG	232 (81.69)	33 (76.74)	0.396	
AA	1 (0.35)	1 (2.33)		
GA	51 (17.96)	9 (20.93)		
G	515 (90.67)	75 (87.21)	0.314	0.702 (0.351–1.403)
A	53 (9.33)	11 (12.79)		

^∗^ *P* < 0.05. SNP: single-nucleotide polymorphism; HT: Hashimoto's thyroiditis; NC: normal controls; OR: odds ratio; CI: confidence interval.

**Table 5:** Allele and genotype frequencies of IL-22 SNPs with onset age and ophthalmopathy in AITD patients.

SNP	Onset age of AITD patients			Ophthalmopathy		
	≥19 (%)	≤18 (%)	*P* value (OR/95% CI)	Without (%)	With (%)	*P* value (OR/95% CI)
rs2046068						
AA	556 (63.91)	66 (62.86)	0.034^∗^	547 (63.83)	75 (63.56)	0.758
CC	35 (4.02)	10 (9.52)		38 (4.43)	7 (5.93)	
AC	279 (32.07)	29 (27.62)		272 (31.74)	36 (30.51)	
A	1391 (79.94)	161 (76.67)	0.266 (0.824/0.586–1.159)	1366 (79.70)	186 (78.81)	0.752 (0.948/0.679–1.323)
C	349 (20.06)	49 (23.33)		348 (20.30)	50 (21.19)	
rs2227478						
TT	556 (63.91)	66 (62.86)	0.028^∗^	547 (63.83)	75 (63.56)	0.719
CC	34 (3.91)	10 (9.52)		37 (4.32)	7 (5.93)	
TC	280 (32.18)	29 (27.62)		273 (31.85)	36 (30.51)	
T	1392 (80.00)	161 (76.67)	0.257 (0.821/0.584–1.155)	1367 (79.75)	186 (78.81)	0.736 (0.944/0.676–1.318)
C	348 (20.00)	49 (23.33)		347 (20.25)	50 (21.19)	
rs2227485						
CC	287 (32.99)	28 (26.67)	0.421	279 (32.56)	36 (30.51)	0.904
TT	201 (23.10)	26 (24.76)		199 (23.22)	28 (23.73)	
CT	382 (43.91)	51 (48.57)		379 (44.22)	54 (45.76)	
C	956 (54.94)	107 (50.95)	0.273 (0.852/0.640–1.135)	937 (54.67)	126 (53.39)	0.712 (0.950/0.723–1.248)
T	784 (45.06)	103 (49.05)		777 (45.33)	110 (46.61)	
rs1179251						
CC	372 (42.76)	55 (52.38)	0.120	368 (42.94)	59 (50.00)	0.116
GG	107 (12.30)	8 (7.62)		98 (11.44)	17 (14.41)	
CG	391 (44.94)	42 (40.00)		391 (45.62)	42 (35.59)	
C	1135 (65.23)	152 (72.38)	0.039^∗^ (1.397/1.016–1.920)	1127 (65.75)	160 (67.80)	0.534 (1.097/0.820–1.466)
G	605 (34.77)	58 (27.62)		587 (34.25)	76 (32.20)	
rs11611206						
GG	694 (79.77)	88 (83.81)	0.386	696 (81.21)	86 (72.88)	0.023^∗^
AA	11 (1.26)	0 (0)		11 (1.28)	0 (0)	
GA	165 (18.97)	17 (16.19)		150 (17.51)	32 (27.12)	
G	1553 (89.25)	193 (91.90)	0.236 (1.367/0.814–2.296)	1542 (89.96)	204 (86.44)	0.097 (0.711/0.474–1.066)
A	187 (10.75)	17 (8.10)		172 (10.04)	32 (13.56)	

^∗^ *P* < 0.05. SNP: single nucleotide polymorphism; AITD: autoimmune thyroid disease; OR: odds ratio; CI: confidence interval.

**Table 6:** Allele and genotype frequencies of IL-22 SNPs with ophthalmopathy in GD patients and hypothyroidism in HT patients.

SNP loci	Ophthalmopathy in GD			Hypothyroidism in HT		
	Without (%)	With (%)	*P* value (OR/95% CI)	Without (%)	With (%)	*P* value (OR/95% CI)
rs2046068						
AA	348 (66.03)	71 (63.39)	0.611	100 (60.61)	103 (60.23)	0.818
CC	22 (4.18)	7 (6.25)		9 (5.45)	7 (4.09)	
AC	157 (29.79)	34 (30.36)		56 (33.94)	61 (35.68)	
A	853 (80.93)	176 (78.57)	0.418 (0.864/0.606–1.231)	256 (77.58)	267 (78.07)	0.877 (1.029/0.715–1.481)
C	201 (19.07)	48 (21.43)		74 (22.42)	75 (21.93)	
rs2227478						
TT	347 (65.84)	71 (63.39)	0.558	101 (61.21)	103 (60.23)	0.789
CC	21 (3.98)	7 (6.25)		9 (5.46)	7 (4.09)	
TC	159 (30.18)	34 (30.36)		55 (33.33)	61 (35.68)	
T	853 (80.93)	176 (78.57)	0.418 (0.864/0.606–1.231)	257 (77.88)	267 (78.07)	0.952 (1.011/0.702–1.457)
C	201 (19.07)	48 (21.43)		73 (22.12)	75 (21.93)	
rs2227485						
CC	166 (31.50)	34 (30.36)	0.958	65 (39.39)	50 (29.24)	0.139
TT	124 (23.53)	26 (23.21)		36 (21.82)	41 (23.98)	
CT	237 (44.97)	52 (46.43)		64 (38.79)	80 (46.78)	
C	569 (53.98)	120 (53.57)	0.910 (0.984/0.737–1.313)	194 (58.79)	180 (52.63)	0.108 (0.779/0.574–1.057)
T	485 (46.02)	104 (46.43)		136 (41.21)	162 (47.37)	
rs1179251						
CC	221 (41.94)	57 (50.89)	0.045^∗^	69 (41.82)	80 (46.78)	0.173
GG	60 (11.38)	17 (15.18)		24 (14.54)	14 (8.19)	
CG	246 (46.68)	38 (33.93)		72 (43.64)	77 (45.03)	
C	688 (65.28)	152 (67.86)	0.460 (1.123/0.826–1.528)	210 (63.64)	237 (69.30)	0.120 (1.290/0.936–1.778)
G	366 (34.72)	72 (32.14)		120 (36.36)	105 (30.70)	
rs11611206						
GG	425 (80.65)	81 (72.32)	0.036^∗^	128 (77.57)	148 (86.55)	0.086
AA	7 (1.33)	0 (0)		3 (1.82)	1 (0.58)	
GA	95 (18.02)	31 (27.68)		34 (20.61)	22 (12.87)	
G	945 (89.66)	193 (86.16)	0.128 (0.718/0.468–1.102)	290 (87.88)	318 (92.98)	0.024^∗^ (1.828/1.075–3.106)
A	109 (10.34)	31 (13.84)		40 (12.12)	24 (7.02)	

^∗^ *P* < 0.05. SNP: single nucleotide polymorphism; GD: Graves' disease; HT: Hashimoto's thyroiditis; OR: odds ratio; CI: confidence interval.

**Table 7:** Distribution of haplotypes for IL-22 gene polymorphisms in AITD, GD, and HT patients and controls groups.

Haplotypes	AITD	GD	HT	NC	*P* value for association		
					AITD versus NC	GD versus NC	HT versus NC
CATT	887	589	298	794	0.429	0.693	0.294
GACT	662	438	224	573	0.914	0.807	0.841
CCCC	395	247	148	321	0.310	0.783	0.087

AITD: autoimmune thyroid disease; GD: Graves' disease; HT: Hashimoto's thyroiditis; NC: normal controls.
